# Spatial resolution and optical sensitivity in the compound eyes of two common European wasps, *Vespula germanica* and *Vespula vulgaris*

**DOI:** 10.1242/jeb.246670

**Published:** 2024-08-22

**Authors:** Daniel Gutiérrez, Elisa Rigosi, Nicolas Nagloo, David O'Carroll, Eric J. Warrant

**Affiliations:** Lund Vision Group, Department of Biology, Lund University, Sölvegatan 35, Lund, S-22362, Sweden

**Keywords:** Insect vision, Social hymenopteran, Visual cues, Visual field, Visual acuity, Interommatidial angle

## Abstract

*Vespula germanica* and *Vespula vulgaris* are two common European wasps that have ecological and economic importance as a result of their artificial introduction into many different countries and environments. Their success has undoubtedly been aided by their capacity for visually guided hunting, foraging, learning and using visual cues in the context of homing and navigation. However, the visual systems of *V. germanica* and *V. vulgaris* have not received any deep attention. We used electrophysiology, together with optical and anatomical techniques, to measure the spatial resolution and optical sensitivity of the compound eyes of both species. We found that both wasps have high anatomical spatial resolution with narrow interommatidial angles (Δϕ between 1.0 and 1.5 deg) and a distinct acute zone in the fronto-ventral part of the eye. These narrow interommatidial angles are matched to photoreceptors having narrow angular sensitivities (acute zone acceptance angles Δρ below 1.3 deg), indicating eyes of high spatial resolution that are well suited to their ecological needs. Additionally, we found that both species possess an optical sensitivity that is typical of other day-flying hymenopterans.

## Teaser

**Summary:** *Vespula germanica* and *Vespula vulgaris* have eyes of high spatial resolution that are well suited to their ecological needs.

## INTRODUCTION

The German wasp, *Vespula germanica*, and the common wasp, *Vespula vulgaris*, are social wasps native to Europe that were accidentally introduced into the USA and Canada (MacDonald et al., 1980; Akre et al., 1989; Gambino, 1991), Chile and Argentina (Edwards, 1976; Masciocchi et al., 2010), South Africa (Whitehead and Prins, 1975), New Zealand (Thomas, 1960; Clapperton et al., 1989) and Australia (Spradbery, 1973). It has been suggested that the success of *V. germanica* and *V. vulgaris* in colonising different environments is mostly due to their food plasticity (Farji-Brener and Corley, 1998; Lozada and D'Adamo, 2006). These species of European wasps are scavengers and opportunistic predators with a highly diverse diet (Harris, 1991; Beggs et al., 2011; Archer and Penney, 2012; Grangier and Lester, 2012). Adults of these species exhibit a foraging behaviour feeding on carrion, garbage, live arthropods and fruits, and in larval stages, they are fed honeydew by the foraging adults (Akre, 1982; Akre, 1991). Many wasp species use visual (Parrish and Fowler, 1983; Raveret and Richter, 1990) and olfactory cues to locate prey (Raveret et al., 1985; Raveret and Richter, 2000). When exploiting a food source, foraging German wasps learn cues from the environment to retrieve memories related to rewarding stimuli (D'Adamo and Lozada, 2011; Lozada and D'Adamo, 2011). Similar to other social species, foraging individuals of *V. germanica* collect food and return with it back to the nest. In social hymenopterans, finding and remembering food sources, as well as making several trips between the food source and the nest, is a frequent behaviour exhibited by foraging individuals. D'Adamo and Lozada (2007) have shown that *V. germanica* is able to learn visual cues while foraging to find and memorize feeding sites.

The visual behaviours of European wasps suggest that they possess compound eyes of good spatial resolution, similar to those of other social hymenopterans such as honeybees (Rigosi et al., 2017a, 2021). Remarkably, however, the morphology and physiology of their apposition compound eyes have never been studied. Moreover, as in many wasps (but not bees), the eyes of European wasps each possess a broad horizontal cuticular ‘peninsula’ that extends from the anterior frons well into the eye, effectively dividing the eye into dorsal and ventral halves over much of the frontal visual field. Whether these cuticular peninsulas disrupt the frontal visual field of these and other wasps to create a visual ‘blind spot’ is unknown. Given that the honeybee has a pronounced frontal acute zone centred just below the horizon (Rigosi et al., 2017a, 2021), one possibility is that the ventral side of the cuticular peninsula seen in many wasps encompasses a localized region of higher visual acuity, while the ommatidia of the dorsal side are arranged for lower acuity tasks, akin to ‘peripheral vision’ in other species.

To test this hypothesis, we investigated the morphology of the compound eyes of *V. germanica* and *V. vulgaris*, and measured their visual spatial resolution using electrophysiological, histological and optical techniques (e.g. Greiner et al., 2004; Warrant et al., 2004). We also took advantage of recently developed methods for mapping physiological acuity and optical spatial sampling across different regions of the compound eye (Rigosi et al., 2017a, 2021). Our results reveal that the apposition compound eyes of European wasps have high spatial resolution that is well matched to their visual ecology.

## MATERIALS AND METHODS

### Animals

Workers of *Vespula germanica* (Fabricius, 1793) and *Vespula vulgaris* (Linnaeus, 1758) were collected between July and October 2021 at the campus of Lund University. All individuals were worker females and during experiments were kept overnight in a refrigerator at 8°C, and during the day in lab conditions (23–26°C). They were fed twice a week with two teaspoons of honey in a Petri dish.

### Electrophysiology

**Fig. 1.**
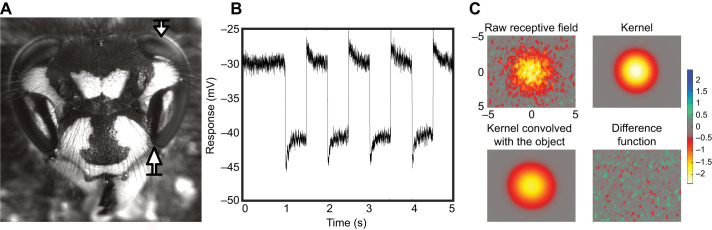
**Electrophysiology.** (A) Location where the electrode was introduced to obtain frontal and lateral photoreceptor recordings (arrows) – shown here in *Vespula vulgaris*. (B) Example of photoreceptor responses to a full-screen flickering stimulus. (C) Electrophysiological responses showing the raw receptive field of a lateral photoreceptor in *V. vulgaris* (5 deg×5 deg). A 2D Gaussian kernel was then fitted to these data to take account of the finite size of the object used to measure it (in this case, a 2.0 deg×2.0 deg black square target). A model fit was obtained by 2D convolution between the estimated kernel and the stimulus at the same spatial scale. Model parameters were iteratively fitted to reduce the error (difference function) between the convolved model and the raw receptive field. Scale indicates membrane voltage in mV.

This procedure followed the protocol of Rigosi et al. (2017a). Individuals of both species were captured and anaesthetised on ice (4°C) for around 20 min. In the meantime, the end of a pipette tube tip was cut off to make a narrow hole. The anaesthetised wasp was inserted into the tube and its head was allowed to emerge through the hole. To avoid any movement during recordings, the thorax, head, pedicel of the antennae and mouthparts were immobilised by waxing them to the pipette using a warm mixture of beeswax and violin rosin (1:1). Next, the wasp was mounted onto a holder and a triangular window (5–10 facets wide) was cut in the dorsal margin of the cornea of the left eye to insert an electrode and permit intracellular recordings within the wasp's left lateral field of view (Fig. 1A). In some cases, the wasp was flipped 180 deg and a hole of the same size was made near the ventral margin of the right compound eye, allowing recordings from the fronto-ventral part of the eye. After the hole was cut, it was covered with silicone grease to prevent the inner ocular tissues from drying out. A reference electrode of thin silver/silver chloride wire was inserted into the other eye. Intracellular membrane potentials of single photoreceptors were recorded using an amplifier with a low-noise, high-input impedance headstage (NPI BA-03X). Sharp electrodes were fabricated from aluminosilicate glass capillaries (SM100F-10, Harvard Apparatus) pulled on a Sutter Instruments P-87 puller and filled with 1 mol l^−1^ KCl solution. Electrode resistance was 80–240 MΩ. A Piezo-controlled manipulator (Marzhauser-Wetzlar, PM-10) allowed the electrode to be stepped through the retina in steps of 3–7 μm. Successful penetration of photoreceptors was revealed by large amplitude (>10 mV) modulation of the membrane potential in response to full screen flicker stimuli, with depolarisation in response to brightening events (Fig. 1B). In total, 50 photoreceptors were used in the analysis for the two species, 24 for *V. germanica* and 26 for *V. vulgari*, in eight individuals for each species.

### Receptive field scans and angular sensitivity estimation of single photoreceptors

One black bar (5 deg×144 deg, velocity 96 deg s^−1^) was presented against a white background on the stimulus screen (Asus VG279QM, resolution 1920×1080 pixels at 280 Hz), positioned 20 cm in front of a wasp facing the screen. The stimulus bar was generated using custom-written software in Matlab (MathWorks, Natick, MA, USA) and it was moved across the screen along the four cardinal directions (left, right, up, down). In response to the movement of a black bar across its receptive field (RF), the photoreceptor experienced a dimming that resulted in a hyperpolarisation of its membrane potential. These responses gave us an estimate of the centre coordinates of the photoreceptor's RF on the screen and allowed us to generate a stimulus sequence around its centre to accurately measure the receptive field of the photoreceptor. A 6 deg square region of interest (ROI) was centred on the RF and a small black square (Weber contrast=−0.998) was drifted at a velocity of 48 deg s^−1^ left to right or from top to bottom within the ROI along 61 sequential raster lines (i.e. with a scanning resolution of 0.15 deg). With a pre-stimulus recording time of 0.5 s the total scanning time required to obtain a complete RF was approximately 120 s, with this sequence repeated while the recording remained stable. The data obtained fed a model that allowed us to fit the measured receptive fields by convolving a 2D Gaussian kernel (Fig. 1C) with an image of the size and shape of the target, rendered at the same resolution as the raster plot. This convolution is required to account for the interaction between actual photoreceptor optical sampling (i.e. image blur) and the finite size of the stimulus target: if the target is large enough, the apparent receptive field would include a ‘neural image’ of the partially resolved target (O'Carroll and Wiederman, 2014). The summed square of the difference (Fig. 1C, lower right) between the convolved image and the observed data was then minimised using a simplex search that varied the *xy* location of the Gaussian kernel, its standard deviation and its amplitude. Horizontal and vertical photoreceptor acceptance angles (Δρ, in degrees) were then estimated from the best-fit kernel (we report the vertical component as the acceptance angle). The same model also allowed us to identify the exact centre coordinates of the RF for subsequent experiments. In fitting a 2D Gaussian (linear) kernel (O'Carroll and Wiederman, 2014), we assume linearity in the measured signal, despite the potential recruitment of voltage-gated conductances and other non-linearities. We therefore selected a target size for the black object that was small enough to limit maximum responses to below 5.5 mV, which we previously showed maintains reasonable linearity in photoreceptor recordings from honeybee foragers (Rigosi et al., 2017a). For frontal recordings, this stimulus target was 1.5 deg×1.5 deg or 2.0 deg×2.0 deg, while for recordings from the lateral field of view the target was 3.0 deg×3.0 deg.

### Receptive field location

For each photoreceptor recorded, the coordinates of the receptive field location on the eye were measured. The distance from the central midpoint in the frontal visual field (0 deg longitude, 0 deg latitude), and perpendicular to the dorsal head axis, was measured in pixels on the stimulus screen and was transformed later into degrees.

### Spatial resolution: optical measurements of interommatidial angle

To measure spatial resolving power in different regions of an apposition compound eye, a well-known method is to use the principal pseudopupil (a small region of ommatidial facets directed towards the viewer that appears as a dark spot on the surface of the eye) to determine local ommatidial density and thus local interommatidial angle Δϕ (Land and Eckert, 1985). By using the pseudopupil to systematically determine ommatidial density at different locations in the eye, one can map variations in interommatidial angle across the visual field, as has been done in a large number of species (e.g. blowflies: Land and Eckert, 1985; Dahmen, 1991; Petrowitz et al., 2000; Straw et al., 2006; bees: Warrant et al., 2004; Rigosi et al., 2017a; mantids: Rossel, 1979; butterflies: Rutowski and Warrant, 2002; Straw et al., 2006).

It was previously only possible to use the pseudopupil method in insects with clear (lightly pigmented) eyes to visualise the dark principal pseudopupil. However, in insects with heavily pigmented eyes (typical of ants, wasps and many bees), the pseudopupil is not visible. It can sometimes be visualised using anti-dromic illumination (directed from beneath the basement membrane of the eye), although this method is difficult and not always successful in heavily pigmented eyes (Spaethe and Chittka, 2003). In the case of dark-eyed European wasps, we overcame these problems by mapping interommatidial angles using a new method that reveals the pseudopupil via the application of fluorescent dyes (Rigosi et al. 2021).

**Fig. 2.**
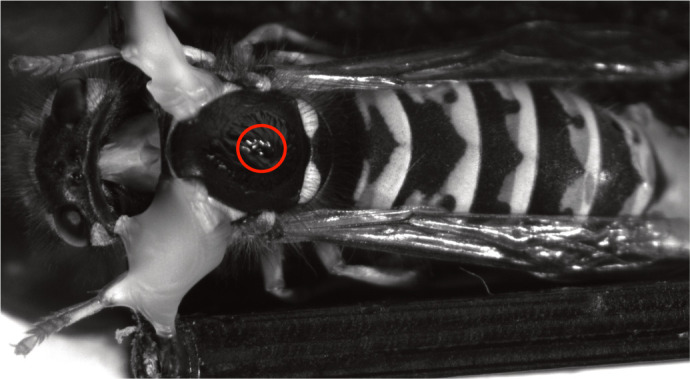
**Thoracic injection site for Lucifer Yellow in *Vespula germanica.*** The fluorescent dye was injected at 1.5 mm depth into the thorax (red circle) to reach the haemocoel and move into the head via the haemolymph.

Female wasps (*N*=1 per species) were anaesthetised using CO_2_ and then mounted on custom-made 3D-printed holders. While the individual was sedated, it was immobilised by waxing the abdomen, wings, thorax, mouthparts and antennae with the same warm wax mixture used for electrophysiological experiments (beeswax and violin rosin 1:1). The method described by Rigosi et al. (2021) was followed with slight modifications. Rather than applying a fluorophore crystal directly within the head, 4 µl of Lucifer Yellow dye (6% concentration in distilled water) was injected at 1.5 mm depth into the thorax using a 100 µl Hamilton syringe fitted to a micro-syringe pump (KD Scientific LEGATO111), at a flow rate of 1 µl min^−1^. This allowed the fluorescent Lucifer Yellow dye to reach the haemocoel and move into the head via the haemolymph (Fig. 2), where it was then taken up by photoreceptors. This then allowed visualisation of the luminous pseudopupil (the small region of ommatidia on the eye surface directed towards the viewer). Once the injection was complete, animals were mounted in a custom-built goniometer constructed from two motorised precision rotation stages (KPRMTE/M, Thorlabs Inc., Newton, NJ, USA) on a manual translation stage (all components from Thorlabs Inc.), using a custom-made 3D printed holder that allowed the subject to rotate around its own axis.

The holder, together with the goniometer, allowed continuous or stepwise rotation of the animal across both longitude (azimuth) and latitude (elevation), rotating about the yaw axis at different elevations as well as rotating in the anterior–posterior (roll) axis. We were able to obtain pictures at up to 90 deg latitude and 100 deg longitude. After the animal was placed on the goniometer, Lucifer Yellow dust was sprinkled on the wasp's eye to create landmarks over the eye surface (used to identify the same ommatidia in sequential photographs, as explained below).

At each 10 deg step of latitude and longitude, an image of the eye surface, including the luminous pseudopupil and landmarks, was captured using a Nikon SMZ18 fluorescence stereomicroscope (Nikon, BergmanLabora AB, Danderyd, Sweden). This microscope was modified by a 180 deg reversal of the imaging head and a rotation of the objective turret to align the episcopic light source (Sola light engine SM-5-LCR-SB Lumencor^®^, Beaverton, OR, USA) coaxially with the imaging pathway of a cooled sCMOS camera (Andor Zyla 5.5, Oxford Instruments) coupled to NIS Elements AR software (version 4.50, Nikon, BergmanLabora AB).

Using these images, we were able to determine the facet coordinates of the facet located at the centre of the luminous pseudopupil in each image (i.e. at each latitude and longitude in the 10 deg grid across the eye) as well as the average local facet diameter *D*. These facet coordinates were determined after defining *x* and *y* facet rows relative to an ‘origin facet’ located at a latitude and longitude of 0 deg (see Warrant et al., 2004, for a full description of the methods). Using custom-built software (Facet 4.03 for Mac OSX, available from https://github.com/insectvision/Facet), these coordinates were used to calculate the ommatidial density at each location in the eye (in ommatidia per square degree), and thus the local average interommatidial angle Δϕ (deg). These values of Δϕ were plotted on a sphere representing 3D space around the wasp and contour lines were interpolated to connect regions having the same Δϕ. Similar plots were also made for facet diameter *D*.

### Histology

Wasps were immobilised by keeping them at 8°C in a refrigerator for 40 min. Following this, they were inserted into a pipette tip whose end was cut to make a hole narrow enough for only the wasp's head to pass through. The wasp's head was then shaved using the rear edge of a razor blade, and subsequently the head was removed. From there, the mouthparts, antennae and cuticle were removed to keep only the eye, which was later transferred into fixative (a mixture of 2.5% glutaraldehyde and 2% paraformaldehyde in phosphate buffer, pH 7.2–7.5). The heads were fixed for 2–3 h at 4°C before being osmicated (2% OsO_4_ in distilled H_2_O) for 1 h. The heads were subsequently dehydrated in an ethanol series, transferred to propylene oxide and embedded in epoxy resin (Fluka). Frontal, longitudinal and tangential serial sections, 3 μm thick, were cut on a Reichert Ultracut microtome using glass knives. The 3 μm thick section series were placed on microscope slides and flattened on a 60°C hot-plate, after which they were stained with Mallory's borax-Methylene Blue. Colour light-micrographs were captured using a Zeiss Axiophot microscope with a Nikon DS-Fi1c camera and NIS-Element D4.20.01 software. For transmission electron microscopy (TEM), ultrathin sections (50 nm) were cut on a Leica EM UC7 microtome with a diamond knife. Sections from the distal rhabdom were mounted on Formvar-coated grids and stained with 6% uranyl acetate (25 min) and 3% lead citrate (10 min), and photographed with a JEOL 1400 PLUS I transmission electron microscope (sCMOS sensor camera) with an accelerating voltage of 100 kV.

Head metrics and corneal facets were studied by scanning electron microscopy (SEM). Air-dried head specimens from female wasps were carefully mounted onto SEM stubs, and sputter-coated with gold (Cesington 108 auto, 70 s, 20 mA). Following this, samples were viewed and analysed in a Hitachi SU3500 scanning electron microscope.

We used the resulting morphological data to calculate the optical sensitivity of the eye in white light illumination, *S* (in units of µm^2^ sr) (Land, 1981; Warrant and Nilsson, 1998):
(1)

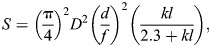
where *D* is the facet diameter (µm), *f* is the focal length of the eye (µm), *d* and *l* are the diameter and length of the photoreceptor (rhabdom), respectively (µm), and *k* is the absorption coefficient of the photoreceptor (taken as 0.0067 µm^−1^: Bruno et al., 1977). This equation allowed us to describe the light capturing capacity of the eye (i.e. image brightness) in wasps compared with other species of insects.

### Image analysis

ImageJ was used to measure the dimensions of internal visual structures (from the acute zone of both species) obtained from the TEM, SEM and light microscopy. In both species (*n*=2 for each species), the diameters (*d*) and lengths (*l*) of five rhabdoms (*n*=5) were measured, while ommatidial length (*L*) was measured 5 times (*n*=5) in *V. germanica* and 7 times (*n*=7) in *V. vulgaris*.

## RESULTS

### General description of the eye

**Fig. 3.**
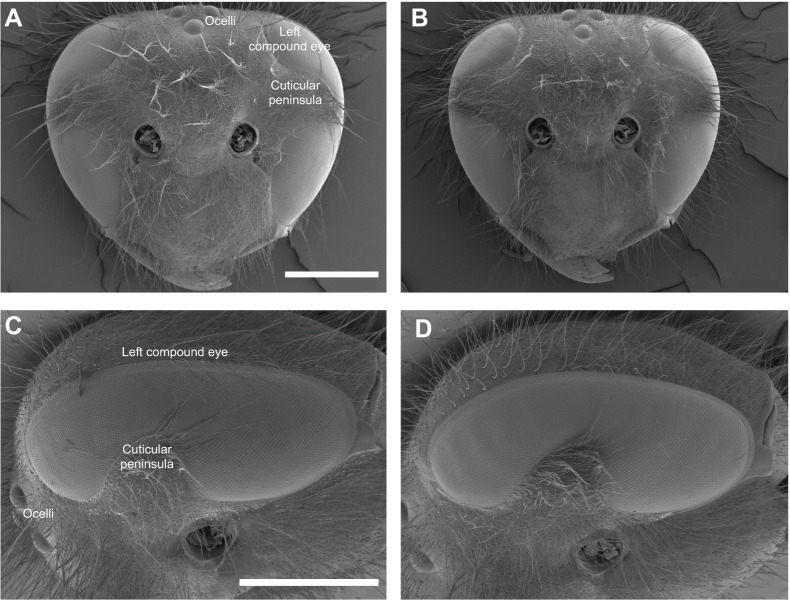
**Scanning electron microscope images of dorso-frontal views of the head (top) and closer lateral images of the eyes (bottom).** (A,C) *Vespula germanica*. (B,D) *Vespula vulgaris*. The antennae have been removed for clarity. Scale bars: 1 mm (scale bar in A also applies to B, and scale bar in C also applies to D).

Both *V. germanica* and *V. vulgaris* have well-formed compound eyes and three ocelli on the dorsal surface of the head (Fig. 3). They also both possess a cuticular ‘peninsula’ that partially divides the eye into a dorsal and ventral part (Fig. 3). It was surmised that this cuticular indentation might create a ‘blind spot’ in the frontal visual field; however, as we will see below, this was not the case.

### Internal eye morphology and optical sensitivity

**Fig. 4.**
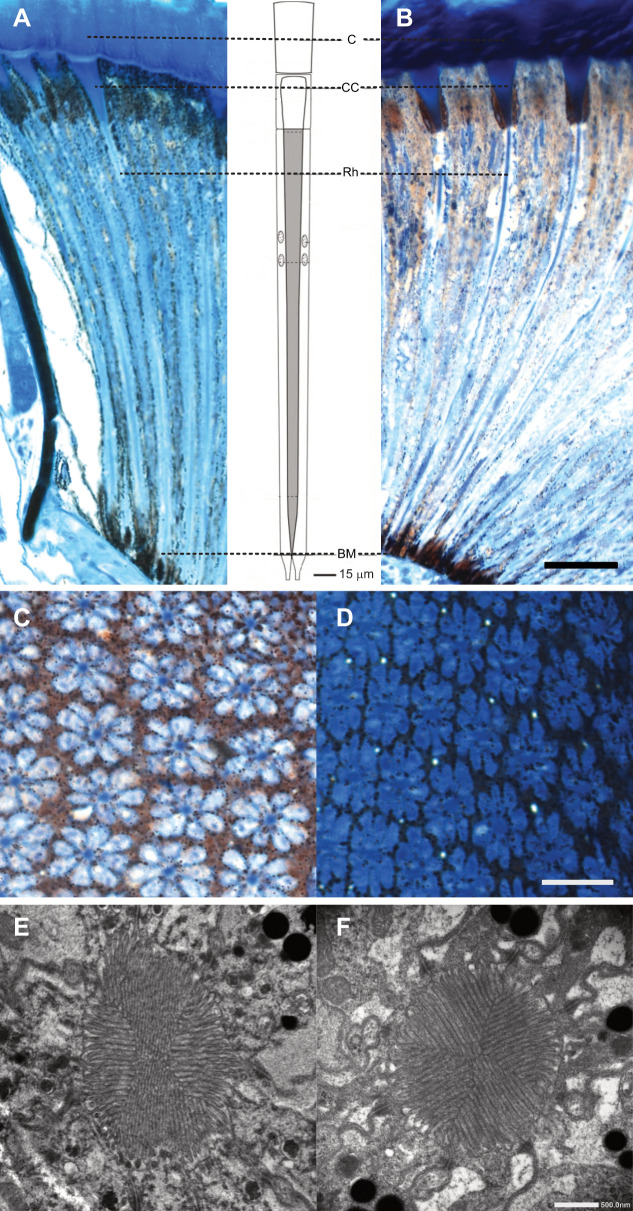
**Internal eye morphology.** (A,B) Longitudinal sections through the apposition compound eyes of female *V. germanica* (A) and *V. vulgaris* (B) with a semi-schematic drawing of an ommatidium (centre, adapted from Greiner et al., 2004). C, corneal facet; CC, crystalline cone; Rh, fused rhabdom; BM, basement membrane. Scale bar: 50 µm (scale bar in B also applies to A). (C,D) Transverse sections of ommatidia in female *V. germanica* (C) and *V. vulgaris* (D). Scale bar: 50 µm (scale bar in D also applies to C). (E,F) Transmission electron micrographs of cross-sections through the distal rhabdoms of *V. germanica* (E) and *V. vulgaris* (F). Scale bar: 500 nm (scale bar in F also applies to E).

**Fig. 5.**
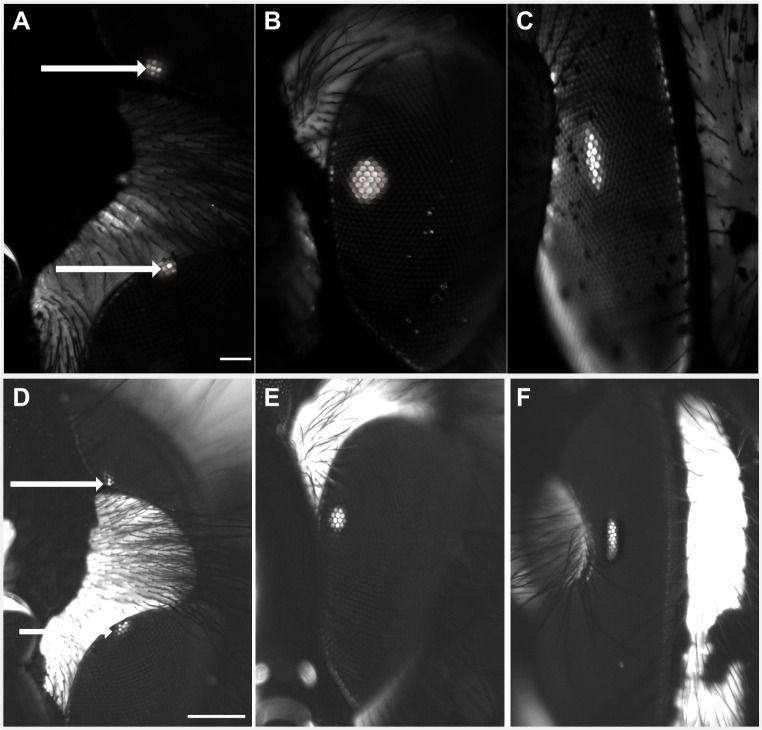
**Luminous pseudopupil in *V. germanica* (top) and *V. vulgaris* (bottom).** (A,D) The pseudopupil is present in both dorsal and ventral ‘halves’ of the eye simultaneously at 0 deg longitude and 0 deg latitude (arrows). (B,E) The pseudopupil in the fronto-ventral zone where the acute zone was found (30 deg longitude, −10 deg latitude). (C,F) The pseudopupil is found at the lateral side of the eye (100 deg longitude, 0 deg latitude). Scale bars: 100 µm (scale bar in A also applies to B and C; scale bar in D also applies to E and F).

The eyes of both wasp species are classical afocal apposition eyes (Fig. 4). The length *l* of the rhabdom, in the frontal acute zone (see below), in *V. germanica* was 227±2 µm, and in *V. vulgaris* it was 255±2 µm (means±s.d.) (Fig. 4A,B). We found that the pseudopupils of these species are divided by the cuticular peninsula, with both the dorsal and ventral eye regions revealing a portion of the pseudopupil simultaneously at the upper and lower edges of the peninsula when viewed from the front at approximately 0 deg of latitude (i.e. when viewed equatorially; Fig. 5A,D). This suggests that rhabdoms on the dorsal and ventral sides of this peninsula view a similar point in space, implying that no blind spot is caused by the cuticular peninsula.

**Fig. 6.**
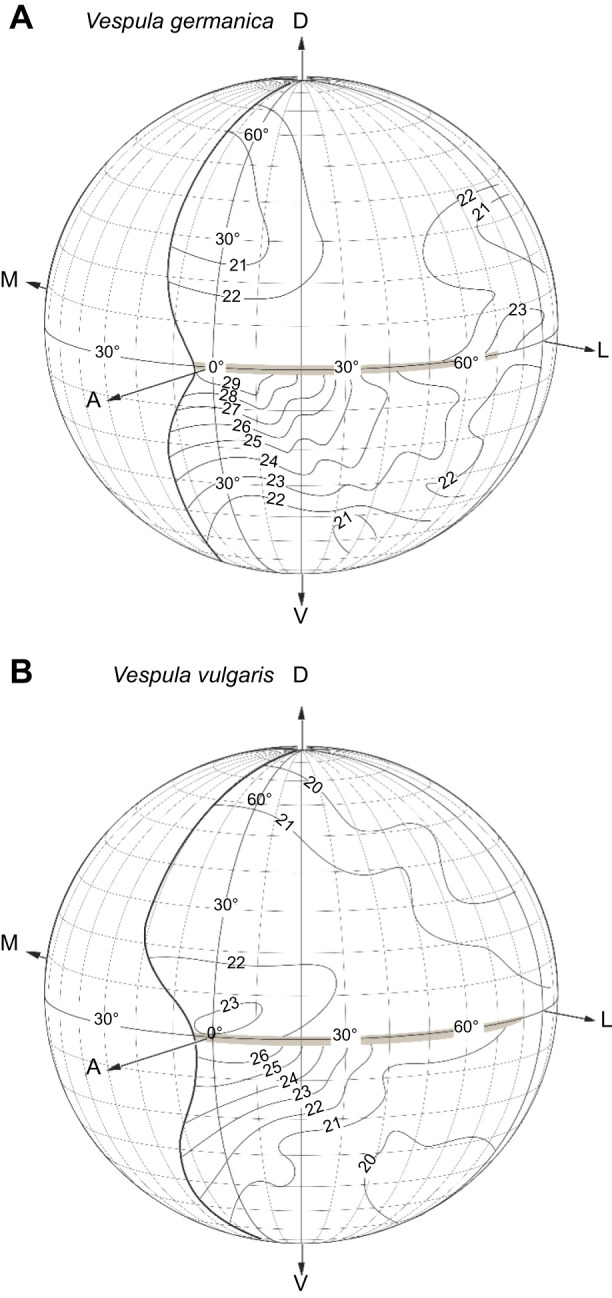
**Facet diameters (*D*, in µm) in the left eye of a female worker.** (A) *Vespula germanica*. (B) *Vespula vulgaris*. Data are plotted as isolines onto a sphere that represents the 3D space around the wasp. Lines of latitude and longitude are shown in intervals of 10 deg. The boundary of the eye's visual field is also shown (thick black line)*.* D, dorsal; V, ventral; A, anterior; M, medial; L, lateral. The grey region centred on 0 deg latitude represents the location of the ‘peninsula’ of cuticle that penetrates the frontal eye but which does not create a ‘blind spot’ within the visual field (it does, however, divide the eye into dorsal and ventral halves with differing spatial properties).

Both species have the largest facet diameter *D* just ventral to the cuticular peninsula, in the fronto-ventral zone of the eye (Fig. 6). From there, the facet diameter decreases smoothly towards the ventral region of the eye. Likewise, in the dorsal part of the eye, facet diameter decreases smoothly frontally to dorsally. Although the two species are very similar, *V. germanica* has larger facet diameters (29 µm) while the largest facet diameters found in *V. vulgaris* were around 26 µm (Fig. 6).

Transmission electron micrographs of transverse sections through the rhabdoms from this frontal eye region reveal the diameter of the rhabdoms *d* (Fig. 4E,F) to be 2.1±0.3 µm in *V. germanica* and 1.9±0.3 µm in *V. vulgaris*. The focal length of the ommatidium *f* has previously been measured for *V. vulgaris* (Kelber et al., 2011) and was found to be 67 µm. We assume this value for both wasp species here.

With these parameters, we were able to calculate the optical sensitivity *S* of the eyes in white light (Eqn 1) at the centres of the acute zone for both species (where facet diameter *D* is 29 µm for *V. germanica* and 26 µm for *V. vulgaris*; Fig. 6). Calculated values of *S* were 0.20 µm^2^ sr for *V. germanica* and 0.14 µm^2^ sr for *V. vulgaris*.

### Anatomical resolving power

In a compound eye, the packing density of ommatidia is inversely proportional to the angle between adjacent ommatidia. This is also known as the interommatidial angle Δϕ, which determines the anatomical spatial resolution of the compound eye (Land, 1981): the smaller the value of Δϕ, the greater the potential resolution. In general, the size of the pseudopupil is a rough indicator of relative resolution, with a larger pseudopupil revealing an eye region having a smaller Δϕ*.* In both wasp species, the largest pseudopupil was observed in the frontal eye region approximately 10 deg ventral to the cuticular peninsula (Fig. 5B,E). At this location, the pseudopupil is symmetrical, implying isotropic resolution in both horizontal and vertical dimensions. As observed in other Hymenoptera (Stavenga, 1979), the pseudopupil becomes noticeably narrower horizontally in lateral regions of the visual field, implying a sharp reduction in horizontal resolution (Fig. 5C,F).

**Fig. 7.**
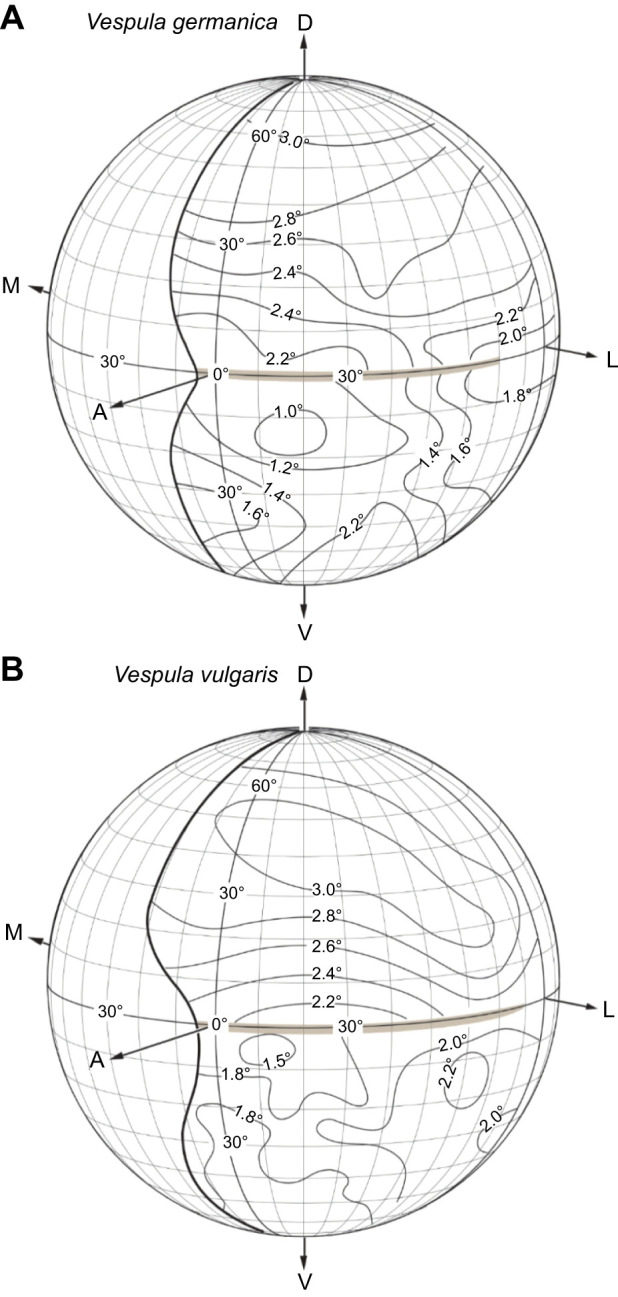
**Interommatidial angles (Δϕ, in degrees) in the left eye of a female worker.** (A) *Vespula germanica*. (B) *Vespula vulgaris*. All other figure conventions as in Fig. 6.

Corresponding to these observations, we found that the local Δϕ (averaged interommatidial angle) was smallest in the fronto-ventral part of the eye in both wasps, approximately 1.0 deg for *V. germanica* between latitudes of −10 deg and −20 deg, and approximately 1.5 deg for *V. vulgaris* between latitudes of 0 deg and −10 deg (Fig. 7). Thus, both wasps possess an ‘acute zone’, a region of the eye (and thus visual field) having greatest spatial resolution. In both wasps, the acute zone is positioned in the frontal visual field, just below the horizontal equator. Beneath this acute zone, Δϕ increased smoothly towards the most ventral part of the eye. The averaged interommatidial angle in the dorsal part of the eye (as delineated by the cuticular ‘peninsula’ dividing the eye), behaved differently from the ventral part of the eye (Fig. 7). The ‘peninsula’ was aligned with the horizontal equator and we found that in both species, Δϕ was greater in the dorsal part of the eye than in the ventral part, i.e. there is a sharp discontinuity with lower resolution just above the visual horizon. The smallest values of Δϕ found in the dorsal eye were almost twice as large as the smallest Δϕ found in the acute zone of the ventral eye (2.0 deg for *V. germanica* and 2.2 deg for *V. vulgaris* between latitudes of 0 deg and +10 deg in both species). From there, Δϕ increased smoothly towards the dorsal region of the eye, increasing to around 3.0 deg in both species.

### Photoreceptor spatial receptive fields

**Fig. 8.**
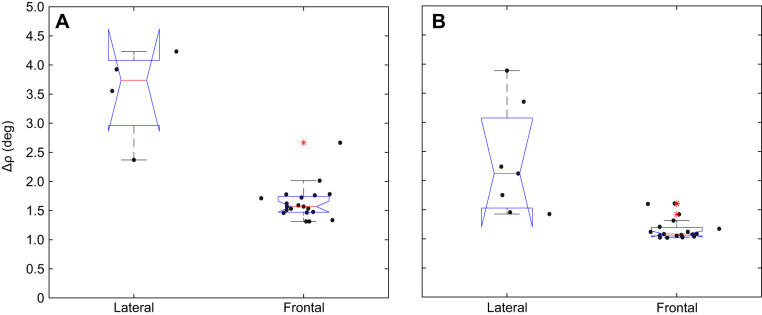
**Acceptance angles (Δρ) for lateral and frontal photoreceptors.** (A) *Vespula germanica*. (B) *Vespula vulgaris*. Data points are scattered along the *x*-axis so they are easier to see. Means of each dataset are represented by a red line and the bottom part of the boxplot corresponds to 25% of the data while the upper part corresponds to 75%. The whiskers extend to the most extreme data points not considered outliers. The outliers are represented by red asterisks. The boxplots are drawn with confidence interval notches; they fold over where these confidence intervals exceed the interquartile range of the data. This is generally because of a small sample size and/or high variability. We used 24 measurements of acceptance angle for *V. germanica* (four for lateral photoreceptors and 20 for frontal photoreceptors) and 26 for *V. vulgaris* (six for lateral photoreceptors and 20 for frontal samples) from eight individuals in each species.

Although we attempted to sample cells from a range of latitudes and longitudes, the overall variability in both recording location and receptive field width (i.e. acceptance angle Δρ) was too large to allow us to directly generate a complete map of Δρ in either the frontal or lateral regions of the eye. Nevertheless, aggregating the data from the frontal and lateral regions (Fig. 8) confirmed the higher acuity estimated optically for the frontal acute zone, with a median Δρ of 1.59 deg (averaged for both species, 95% confidence intervals at 1.48 deg and 1.69 deg, *n*=39; Fig. 8A). The smallest values of Δρ were located primarily around a region between longitudes of 0 and 5 deg (with 9 of the smallest 10 values recorded in this area) and between latitudes of −10 and −20 deg (with 7 of the 10 smallest values recorded in this area), corresponding to the region of the eye with the smallest interommatidial angles (Fig. 7). By comparison, the 11 cells recorded laterally revealed receptive fields almost twice as wide, with a median Δρ of 2.82 deg (Figs 7 and 8).

**Fig. 9.**
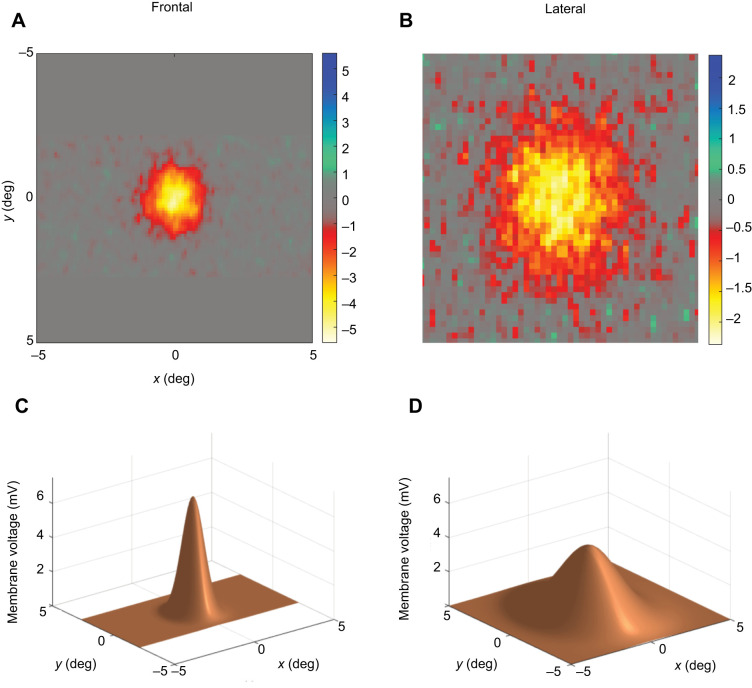
**Comparison of receptive fields obtained from frontal (left, Δρ=1.18** **deg) and lateral (right, Δρ=1.73** **deg) photoreceptors in the eyes of *V. vulgaris*.** (A,B) Raw receptive field data obtained from frontal and lateral photoreceptors where the difference in receptive field size is clearly apparent. *x* and *y* are shown in degrees while the colour bar represents the different membrane voltages in millivolts. (C,D) 2D Gaussian kernels that provide the best fit to the receptive field (as described in Fig. 1C and in Materials and Methods).

**Fig. 10.**
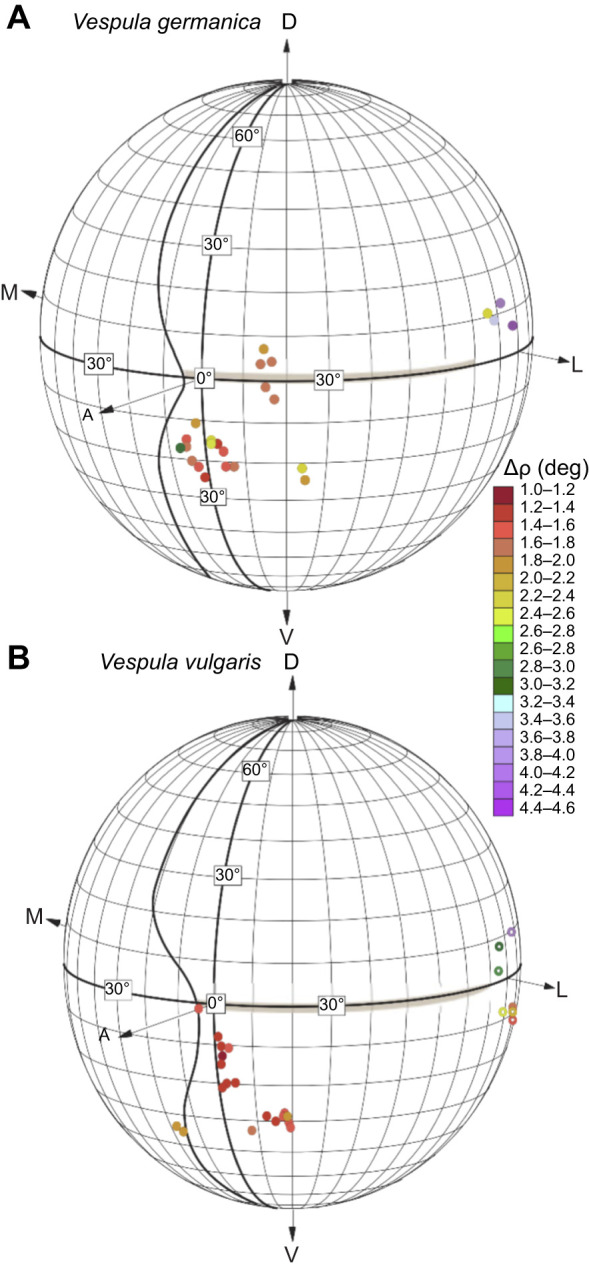
**Receptive field widths (Δρ, in degrees) in the left eye.** (A) *Vespula germanica*. (B) *Vespula vulgaris*. All conventions as in Fig. 6. Open circles represent receptive fields located at a longitude greater than 100 deg, on the rear-side of the globe.

We also obtained several individual recordings from cells from the frontal eye region that could be held long enough to scan the receptive field several times, thereby obtaining high-quality receptive field maps, with Δρ below 1.3 deg (Figs 8 and 9). A comparison of these values in the two wasp species reveals that they are very similar (as shown by the plots in Fig. 8A,B, and by the individual cell acceptance angle Δρ values plotted on the globe in Fig. 10): the sharpest values of Δρ are found frontally and the coarser values are found laterally (Fig. 9).

## DISCUSSION

In the compound eyes of insects, the angular density of the ommatidia (inversely proportional to the interommatidial angle, Δϕ) defines the anatomical resolution of the eye. If an individual has smaller Δϕ, it will potentially have greater spatial resolution (Land, 1981, 1999). A possible complicating factor is that these and many other wasps possess an equatorial cuticular ‘peninsula’ that partially divides their compound eyes into dorsal and ventral halves. Even though this peninsula potentially could have created a blind spot in the frontal visual field, we discovered that this was not the case in the two wasps we studied. Thus, this cuticular peninsula creates a division between the dorsal region of the eye with lower visual acuity and the ventral region with higher visual acuity. Interestingly, however, the way that interommatidial angles varied within the eye changed abruptly at this peninsula, with the dorsal half of the eye generally having higher Δϕ and lower spatial resolving power than the ventral half of the eye (Fig. 7). Why this peninsula is a common feature in the eyes of wasps remains unknown.

In general, we found that the average interommatidial angle in wasps decreases smoothly from the dorsal part of the eye to the equator (i.e. from 80 deg to 0 deg) and increases smoothly again towards the ventral part of the eye (latitude from 0 deg to −80 deg). Although both species exhibited small minimum Δϕ values (Fig. 7), *V. germanica* showed the smallest average Δϕ of 1.0 deg, at around −10 deg latitude and between 10 and 20 deg in longitude. Although *V. vulgaris* has the larger minimum values of Δϕ, they are found in an eye region similar to that in *V. germanica*. These small values of Δϕ (Fig. 7), together with larger facets (Fig. 6) and narrower photoreceptor receptive fields at the same location (Figs 7–9), indicate the presence of an ‘acute zone’ that allows higher spatial resolution in the fronto-ventral part of the eye. The acute zones of these wasps afford a visual performance rivalling the best seen performance in other day-flying hymenopterans, such as the carpenter bees *Xylocopa leucothorax* and *Xylocopa tenuiscapa* (Somanathan et al., 2009a; Somanathan et al., 2009b) and the hornet *Vespa crabro* (Kelber et al., 2011).

A drawback of our method for determining interommatidial angle – which is derived from a local average of ommatidial density – is that it fails to capture possible differences in vertical and horizontal interommatidial angle (and thus spatial resolution) in regions of the eye where corneal surface radius differs significantly in the vertical and horizontal directions. This is a particular issue in the eyes of many bees and wasps, which tend to be more elliptical in shape (Stavenga, 1979), rather than more spherical as is common in butterflies and moths. In areas of the eye where this issue becomes problematic, the luminous pseudopupil becomes more elliptical in shape. In European wasps, this occurs in more lateral regions of the eye (Fig. 5C,F), suggesting that in these regions the interommatidial angle is smaller in the vertical direction than in the horizontal direction. Unfortunately, this difference is not captured by the method we employed. In the frontal visual field, the luminous pseudopupil is more circular, indicating small differences in vertical and horizontal interommatidial angles – here our method works well.

If we compare the photoreceptor receptive fields of *V. germanica* and *V. vulgaris* with those of other insects, it is evident that these wasps have reasonably small values of acceptance angle Δρ, and thus high spatial resolution. Worker honeybees (*Apis mellifera*) have a frontal acute zone within which photoreceptors have an average acceptance angle of 1.6 deg (Rigosi et al., 2017a), while other species such as the drone fly *Eristalis tenax* (Δρ=0.9 deg) (Rigosi et al., 2017b), the butterfly *Melanitis leda* (Δρ=1.5 deg) (Land and Osorio, 1990) and the flies *Calliphora erythrocephala* (Δρ=1.0 deg) (Smakman et al., 1984) and *Calliphora stygia* (Δρ=0.9 deg) (Rigosi et al., 2017b) have similar frontal acceptance angles and consequently similar spatial resolution to *V. germanica* and *V. vulgaris*.

The size of the acceptance angle Δρ is determined by two optical properties of the ommatidium (Snyder, 1979): (1) the quality of the optical image focused on the rhabdom (which in a diffraction-limited eye is equivalent to the angular half-width of the Airy disk, λ/*D* radians, where *D* is the facet diameter and λ is the wavelength of incident light), and (2) the geometrical angular size of the rhabdom (*d*/*f* radians, where *d* is the rhabdom diameter and *f* is the focal length). In a light-adapted apposition compound eye, when pupillary pigments tightly ensheath the rhabdom, the acceptance angle is dominated by the half-width of the Airy disk (Stavenga, 2003a,b; 2004a,b). If we consider the ventro-frontal acute zone of *V. germanica*, *D*≈26 µm (Fig. 6A). If we assume incident green light of wavelength 530 nm (i.e. λ=0.53 µm), the half-width of the Airy disk (λ/*D*) is 0.020 radians, or 1.17 deg. This matches the actual values of Δρ measured in the same part of the eye quite well, where we recorded reliable values below 1.3 deg (Fig. 7A).

Insects with Δρ less than 1 deg are often predators, including dragonflies (Laughlin, 1974), mantids (Rossel, 1979), killer flies (Wardill et al., 2017) and some wasps (Kelber et al., 2011). These insects typically have a forward pointing acute zone for tracking prey (Land, 1997). Even though Δρ (and Δϕ) are larger than 1 deg in wasps, *V. germanica* and *V. vulgaris* are nonetheless predators (and scavengers) that seek different types of protein sources (Broekhuizen and Hordijk, 1968; Archer, 1977; Gambino, 1986; Spradbery, 1973; Spradbery,1991; Edwards, 1980), including flies, lepidopteran larvae, ants, grasshoppers and arachnids (Harris, 1991). Both species hunt from above, and their fronto-ventral acute zones would thus be beneficial for this purpose (Harris et al., 1991; D'Adamo and Lozada, 2007). These two species are also known to hunt continuously, seeking food and returning with it to the larvae and individuals that stay in the nest (these species do not store food in the hexagonal chambers of the nest: Reeve and Gamboa, 1983). It has been reported (Harris et al., 1991) that in forests where *V. germanica* and *V. vulgaris* co-exist, the former forages more on the forest floor and in open areas surrounding the forest while the latter tends to forage more around the foliage of trees and shrubs (Harris et al., 1991). Floral nectar is also an important part of the diet of these wasps and their acute zones are well positioned to view flowers as the wasps land. Additionally, *V. germanica* and *V. vulgaris* are ground-nesting wasps and the nest entrance is well positioned within the acute zone during landing.

The acute zones of *V. germanica* and *V. vulgaris* are similar to those found in the cathemeral European hornet (*V. crabro*). As in the wasps studied here, this species possesses an acute zone in the fronto-ventral region of the eye, with narrower interommatidial angles and larger facets (Kelber et al., 2011). These similarities between *V. germanica*, *V. vulgaris* and *V. crabro* can be explained by examining their biology and predatorial behaviour. All three species are predators that hunt from above, in some cases hovering over the prey. However, while *V. germanica* and *V. vulgaris* build their nests at ground level, *V. crabro* builds its nests in higher places. In contrast, the nocturnal sweat bee (*Megalopta genalis*) has an acute zone aligned along the frontal equator of the eye (Warrant et al., 2004), a location well suited to the landing behaviour of *M. genalis* at its nest.

Not all insects with acute zones have evolved them for predation or landing. Some have evolved them in the context of sex. For instance, the males of higher flies (such as *Calliphora vicina*, *Musca domestica* and *Eristalis tenax*) have larger eyes than females and exhibit an acute zone in the fronto-dorsal region of the eye (Land, 1985; Van Hateren et al., 1989; Straw et al., 2006). The females lack such an acute zone. Male flies use this acute zone to chase females for mating and to chase males during territorial fights (Straw et al., 2006). Some butterflies also exhibit such sexual dimorphism in eye design in the context of mating (e.g. *Asterocampa leila*; Rutowski and Warrant, 2002). However, whether sexual dimorphism is evident in the eyes of *V. germanica* and *V. vulgaris* workers, queens and drones has not been investigated.

Our estimated optical sensitivity *S* of the eyes of the two wasps at the centre of their acute zones (0.20 µm^2^ sr for *V. germanica* and 0.14 µm^2^ sr for *V. vulgaris*) is in the range that is typical of diurnal insects with apposition compound eyes (Land, 1997). Indeed, these values are very close to those estimated for other diurnal hymenopterans with apposition eyes: for example, the carpenter bees *X. tenuiscapa* and *X. leucothorax* (*S*=0.3 and 0.1 µm^2^ sr, respectively: Somanathan et al., 2009a), the honeybee *Apis mellifera* (*S*=0.1 µm^2^ sr: Greiner et al., 2004) and the wasp *Polistes occidentalis* (*S*=0.1 µm^2^ sr: Greiner, 2006).

In conclusion, *V. germanica* and *V. vulgaris* have compound eyes with good spatial acuity. They each possess an acute zone in the ventro-frontal part in the eye, with high spatial resolution subserved by small interommatidial angles and narrow photoreceptor receptive fields (i.e. small acceptance angles), optical properties well suited to the hunting and foraging lifestyles that characterise these two species of wasps.

## Diversity & Inclusion

One or more of the authors of this paper self-identifies as an underrepresented minority in science.

## References

[JEB246670C1] Akre, R. (1982). Social wasps. *Social Insects* 4, 1-105.

[JEB246670C2] Akre, R. (1991). Wasp research: strengths, weaknesses, and future directions. *N. Z. J. Zool.* 18, 223-227. 10.1080/03014223.1991.10757970

[JEB246670C3] Akre, R., Ramsay, C. A. and Grable, A. (1989). Additional range extension by the German Yellowjacket *Paravespula gemanica* (Fabricius), in North America. *Pan-Pac. Entomol.* 65, 79-88.

[JEB246670C4] Archer, M. E. (1977). The weights of forager loads of *Paravespula vulgaris* (Linn.) (Hymenoptera: Vespidae) and the relationship of load weight to forager size. *Insect sociax* 24, 95-102. 10.1007/BF02223281

[JEB246670C5] Archer, M. E. and Penney, D. (2012). *Vespine Wasps of the World: Behaviour, Ecology And Taxonomy of the Vespinae*. Castleton, UK: Siri Scientific Press.

[JEB246670C6] Beggs, J. R., Brockerhoff, E. G., Corley, J. C., Kenis, M., Masciocchi, M., Muller, F., Rome, Q. and Villemant, C. (2011). Ecological effects and management of invasive alien Vespidae. *BioControl* 56, 505-526. 10.1007/s10526-011-9389-z

[JEB246670C7] Broekhuizen, S. and Hordijk, C. (1968). Untersuchungen über die Beute von *Paravespula vulgaris* L. (Hym., Vespidae) und ihre Abhängigkeit von der Beutetierdichte. *Z. Angew. Entomol.* 62, 68-77. 10.1111/j.1439-0418.1968.tb04109.x

[JEB246670C8] Bruno, M. S., Barnes, S. N. and Goldsmith, T. H. (1977). The visual pigment and visual cycle of the lobster, Homarus. *J. Comp. Physiol.* 120, 123-142.

[JEB246670C9] Clapperton, B. K., Möller, H. and Sandlant, G. R. (1989). Distribution of social wasps (Hymenoptera: Vespidae) in New Zealand in 1987. *N. Z. J. Zool.* 16, 315-323. 10.1080/03014223.1989.10422896

[JEB246670C10] D'Adamo, P. and Lozada, M. (2007). Foraging behavior related to habitat characteristics in the invasive wasp *Vespula germanica*. *J. Insect Sci.* 14, 383-388. 10.1111/j.1744-7917.2007.00165.x

[JEB246670C11] D'Adamo, P. and Lozada, M. (2011). Cognitive plasticity in foraging *Vespula germanica* wasps. *J. Insect Sci.* 11, 103. 22221198 10.1673/031.011.10301 PMC3281387

[JEB246670C12] Dahmen, H. (1991). Eye specialisation in waterstriders: an adaptation to life in a flat world. *J. Comp. Physiol. A* 169, 623-632. 10.1007/BF00193552

[JEB246670C13] Edwards, R. (1976). The world distribution pattern of the German wasp, *Paravespula germanica* (Hymenoptera: Vespidae). *Entomol. Germ.* 3, 269-271.

[JEB246670C14] Edwards, R. (1980). *Social Wasps, their Biology and Control*. East Grinstead: The Rentokil Library, 338.

[JEB246670C60] Farji-Brener, A. G., and Corley, J. C. (1998). Successful invasions of hymenopteran insects into NW Patagonia. *Ecol. Austral* 8, 237-249.

[JEB246670C15] Gambino, P. (1986). Winter prey collection at a perennial colony of *Paravespula vulgaris* (L.) (Hymenoptera: Vespidae). *Psyche* 93, 331-340. 10.1155/1986/51785

[JEB246670C16] Gambino, P. (1991). Reproductive plasticity of *Vespula pensylvanica* (Hymenoptera: Vespidae) on Maui and Hawaii Islands, USA. *N. Z. J. Zool.* 18, 139-149. 10.1080/03014223.1991.10757960

[JEB246670C17] Greiner, B. (2006). Adaptations for nocturnal vision in insect apposition eyes. *Int. Rev. Cytol.* 250, 1-46. 10.1016/S0074-7696(06)50001-4 16861062

[JEB246670C18] Grangier, J. and Lester, P. J. (2012). Behavioral plasticity mediates asymmetric competition between invasive wasps and native ants. *Commun. Integr. Biol.* 5, 127-129. 10.4161/cib.18887 22808314 PMC3376045

[JEB246670C19] Greiner, B., Ribi, W. A. and Warrant, E. J. (2004). Retinal and optical adaptations for nocturnal vision in the halictid bee *Megalopta genalis*. *Cell Tissue Res.* 316, 377-390. 10.1007/s00441-004-0883-9 15064946

[JEB246670C20] Gutiérrez, D. (2022). Spatial resolution and optical sensitivity in the compound eyes of two common wasps, Vespula germanica and *Vespula vulgaris*. *MSc thesis*, Linköping University. https://nbid43.ifm.liu.se/2022-2/daniel-gutierrez/10.1242/jeb.246670 PMC11418185 39058380

[JEB246670C21] Harris, R. J. (1991). Diet of the wasps *Vespula vulgaris* and *V. germanica* in honeydew beech forest of the South Island, New Zealand. *N. Z. J. Zool.* 18, 159-169. 10.1080/03014223.1991.10757963

[JEB246670C22] Harris, R. J., Thomas, C. D. and Moller, H. (1991). The influence of habitat use and foraging on the replacement of one introduced wasp species by another in New Zealand. *Ecol. Entomol.* 16, 441-448. 10.1111/j.1365-2311.1991.tb00237.x

[JEB246670C23] Kelber, A., Jonsson, F., Wallén, R., Warrant, E. J., Kornfeldt, T. and Baird, E. (2011). Hornets can fly at night without obvious adaptations of eyes and ocelli. *PLoS ONE* 6, e21892. 10.1371/journal.pone.0021892 21765923 PMC3134451

[JEB246670C24] Land, M. F. (1981). Optics and vision in invertebrates. In *Handbook of Sensory Physiology*, Vol. VII/6B (ed. H. Autrum), pp. 471-592.

[JEB246670C25] Land, M. F. (1997). Visual acuity in insects. *Annu. Rev. Entomol.* 42, 147-177. 10.1146/annurev.ento.42.1.147 15012311

[JEB246670C26] Land, M. F. (1999). Compound eye structure: matching eye to environment. *Adaptive Mechanisms in the Ecology of Vision* (ed. S. N. Archer, M. B. A. Djamgoz, E. R. Loew, J. C. Partridge and S. Vallerga), pp. 51-71. Springer. 10.1007/978-94-017-0619-3_3

[JEB246670C27] Land, M. F. and Eckert, H. (1985). Maps of the acute zones of fly eyes. *Comp. Physiol.* 156, 525-538. 10.1007/BF00613976

[JEB246670C28] Land, M. F. and Osorio, D. C. (1990). Waveguide modes and pupil action in the eyes of butterflies. *Proc. R. Soc. Lond. B Biol. Sci.* 241, 93-100. 10.1098/rspb.1990.0071

[JEB246670C29] Laughlin, S. B. (1974). Neural integration in the first optic neuropile of dragonflies. *Comp. Physiol.* 92, 377-396. 10.1007/BF00694708

[JEB246670C61] Lozada, M. and D'Adamo, P. (2006). How long do *Vespula germanica* wasps search for a food source that is no longer available? *J. Insect Behav.* 19, 591-600. 10.1007/s10905-006-9045-0

[JEB246670C30] Lozada, M. and D'Adamo, P. (2011). Past experience: a help or a hindrance to *Vespula germanica* foragers? *J. Insect Behav.* 24, 159-166. 10.1007/s10905-010-9244-6

[JEB246670C31] MacDonald, J. F., Akre, R. D. and Keyel, R. E. (1980). The German yellowjacket (*Vespula germanica*) problem in the United States (Hymenoptera: Vespidae). *Bull. Entomol. Soc. Am.* 26, 436-444.

[JEB246670C32] Masciocchi, M., Beggs, J. R., Carpenter, J. M. and Corley, J. C. (2010). Primer registro de *Vespula vulgaris* (Hymenoptera: Vespidae) en la Argentina. *Rev. Soc. Entomol. Argent.* 69, 267-270.

[JEB246670C33] O'Carroll, D. and Wiederman, S. D. (2014). Contrast sensitivity and the detection of moving patterns and features. *Phil. Trans. R. Soc. B* 369, 2013004320130043. 10.1098/rstb.2013.0043 PMC3886331 24395970

[JEB246670C63] Parrish, M. D., and Fowler, H. G. (1983). Contrasting foraging related behaviours in two sympatric wasps (*Vespula maculifrons* and *V. germanica*). *Behav. Ecol. Sociobiol.* 12, 25-30. 10.1111/j.1365-2311.1983.tb00497.x

[JEB246670C34] Petrowitz, R., Dahmen, H., Egelhaaf, M. and Krapp, H. G. (2000). Arrangement of optical axes and spatial resolution in the compound eye of the female blowfly *Calliphora*. *J. Comp. Physiol. A* 186, 737-746. 10.1007/s003590000127 11016789

[JEB246670C35] Reeve, H. K. and Gamboa, G. J. (1983). Colony activity integration in primitively eusocial wasps: the role of the queen (*Polistes fuscatus*, Hymenoptera: Vespidae). *Behav. Ecol. Sociobiol.* 13, 63-74. 10.1007/BF00295077

[JEB246670C70] Richter, M. R. and Jeanne, R. L. (1985). Predatory behavior of Polybia sericea (Olivier), a tropical social wasp (*Hymenoptera: Vespidae*). *Behav. Ecol. Sociobiol.* 16, 165-170. 10.1007/BF00295151

[JEB246670C71] Richter, M. R. (1990). Hunting social wasp interactions: influence of prey size, arrival order, and wasp species. *Ecology* 71, 1018-1030. 10.2307/1937370

[JEB246670C59] Richter, M. R. (2000). Social wasp (Hymenoptera: Vespidae) foraging behavior. *Annu. Rev. Entomol.* 45, 121-150. 10.1146/annurev.ento.45.1.121 10761573

[JEB246670C36] Rigosi, E., Wiederman, S. D. and O'Carroll, D. C. (2017a). Visual acuity of the honey bee retina and the limits for feature detection. *Sci. Rep.* 7, 1-7. 10.1038/srep45972 28383025 PMC5382694

[JEB246670C37] Rigosi, E., Wiederman, S. D. and O'Carroll, D. C. (2017b). Photoreceptor signalling is sufficient to explain the detectability threshold of insect aerial pursuers. *J. Exp. Biol.* 220, 4364-4369. 10.1242/jeb.166207 29187619

[JEB246670C38] Rigosi, E., Warrant, E. J. and O'Carroll, D. C. (2021). A new, fluorescence-based method for visualizing the pseudopupil and assessing optical acuity in the dark compound eyes of honeybees and other insects. *Sci. Rep.* 11, 1-12. 10.1038/s41598-020-79139-8 34711871 PMC8553845

[JEB246670C39] Rossel, S. (1979). Regional differences in photoreceptor performance in the eye of the praying mantis. *J. Comp. Physiol.* 131, 95-112. 10.1007/BF00619070

[JEB246670C40] Rutowski, R. L. and Warrant, E. J. (2002). Visual field structure in the Empress Leilia, *Asterocampa leilia* (Lepidoptera, Nymphalidae): dimensions and regional variation in acuity. *J. Comp. Physiol. A Neuroethol. Sens. Neural Behav. Physiol.* 188, 1-12. 11935226 10.1007/s00359-001-0273-7

[JEB246670C41] Smakman, J. G. J., Hateren, J. H. and Stavenga, D. G. (1984). Angular sensitivity of blowfly photoreceptors: intracellular measurements and wave-optical predictions. *J. Comp. Physiol. A* 155, 239-247. 10.1007/BF00612641

[JEB246670C42] Snyder, A. W. (1979). Physics of vision in compound eyes. In *Handbook of Sensory Physiology* (ed. H. Autrum), Vol. VII/6A, pp. 225-313. Springer.

[JEB246670C43] Somanathan, H., Warrant, E. J., Borges, R. M., Wallén, R. and Kelber, A. (2009a). Resolution and sensitivity of the eyes of the Asian honeybees *Apis forea*, *Apis cerana* and *Apis dorsata*. *J. Exp. Biol.* 212, 2448-2453. 10.1242/jeb.031484 19617438

[JEB246670C44] Somanathan, H., Kelber, A., Borges, R. M., Wallén, R. and Warrant, E. J. (2009b). Visual ecology of Indian carpenter bees II: adaptations of eyes and ocelli to nocturnal and diurnal lifestyles. *J. Comp. Physiol. A* 195, 571-583. 10.1007/s00359-009-0432-9 19363615

[JEB246670C45] Spaethe, J. and Chittka, L. (2003). Interindividual variation of eye optics and single object resolution in bumblebees. *J. Exp. Biol.* 206, 3447-3453. 10.1242/jeb.00570 12939375

[JEB246670C46] Spradbery, J. P. (1973). *Wasps. An Account of the Biology and Natural History of Social and Solitary Wasps, With Particular Reference to Those of The British Isles*. Sidgwick & Jackson Ltd.

[JEB246670C47] Spradbery, J. (1991). An orphaned colony of the European wasp *Vespula germanica* (F.) (Hymenoptera: Vespidae) in Australia resulting from repeated usurpation. *N. Z. J. Zool.* 18, 101-103. 10.1080/03014223.1991.10757955

[JEB246670C48] Stavenga, D. G. (1979). Pseudopupils of compound eyes. In *Comparative Physiology and Evolution of Vision in Invertebrates. Handbook of Sensory Physiology*, Vol. VII/6A (ed. H. Autrum), pp. 357-439. Springer. 10.1007/978-3-642-66999-6_7

[JEB246670C49] Stavenga, D. G. (2003a). Angular and spectral sensitivity of fly photoreceptors. I. Integrated facet lens and rhabdomere optics. *J. Comp. Physiol. A* 189, 1-17. 10.1007/s00359-002-0370-2 12548425

[JEB246670C50] Stavenga, D. G. (2003b). Angular and spectral sensitivity of fly receptors. II. Dependence on facet lens F-number and rhabdomere type in *Drosophila*. *J. Comp. Physiol. A* 189, 189-202. 10.1007/s00359-003-0390-6 12664095

[JEB246670C51] Stavenga, D. G. (2004a). Angular and spectral sensitivity of fly receptors. III. Dependence on the pupil mechanism in the blowfly *Calliphora*. *J. Comp. Physiol. A* 190, 115-129. 10.1007/s00359-003-0477-0 14714136

[JEB246670C52] Stavenga, D. G. (2004b). Visual acuity of fly photoreceptors in natural conditions dependence on UV sensitizing pigment and light-controlling pupil. *J. Exp. Biol.* 207, 1703-1713. 10.1242/jeb.00949 15073203

[JEB246670C53] Straw, A. D., Warrant, E. J. and O'Carroll, D. C. (2006). A bright zone in male hoverfly (*Eristalis tenax*) eyes and associated faster motion detection and increased contrast sensitivity. *J. Exp. Biol.* 209, 4339-4354. 10.1242/jeb.02517 17050849

[JEB246670C54] Thomas, C. R. (1960). *The European wasp (Vespula germanica Fab.) in New Zealand*. Cornell University.

[JEB246670C62] Van Hateren, J. H., Hardie, R. C., Rudolph, A., Laughlin, S. B., and Stavenga, D. G. (1989). The bright zone, a specialized dorsal eye region in the male blowfly *Chrysomyia megacephala*. *J. Comp. Physiol. A* 164, 297-308. 10.1007/BF00612990

[JEB246670C55] Wardill, T. J., Fabian, S. T., Pettigrew, A. C., Stavenga, D. G., Nordström, K. and Gonzalez-Bellido, P. T. (2017). A novel interception strategy in a miniature robber fly with extreme visual acuity. *Curr. Biol.* 27, 854-859. 10.1016/j.cub.2017.01.050 28286000 PMC5364399

[JEB246670C56] Warrant, E. J. and Nilsson, D. (1998). Absorption of white light in photoreceptors. *Vision Res.* 38, 195-207. 10.1016/S0042-6989(97)00151-X 9536349

[JEB246670C57] Warrant, E. J., Kelber, A., Gislén, A., Greiner, B., Ribi, W. and Wcislo, W. T. (2004). Nocturnal vision and landmark orientation in a tropical halictid bee. *Curr. Biol.* 14, 1309-1318. 10.1016/j.cub.2004.07.057 15296747

[JEB246670C58] Whitehead, V. and Prins, A. (1975). The European wasp, *Vespula germanica* (F.), in the Cape Peninsula. *Afr. Entomol.* 38, 39-42.

